# Testis-Specific GTPase (TSG): An oligomeric protein

**DOI:** 10.1186/s12864-016-3145-9

**Published:** 2016-10-10

**Authors:** Sudeep Kumar, Hyun Joo Lee, Hee-Sae Park, Keesook Lee

**Affiliations:** 1 Hormone Research Center, School of Biological Sciences and Technology, Chonnam National University, Gwangju, Republic of Korea; 2 Department of Nursing, Dongkang College, Gwangju, Republic of Korea

**Keywords:** GTPase, Testis, RASEF

## Abstract

**Background:**

Ras-related proteins in brain (Rab)-family proteins are key members of the membrane trafficking pathway in cells. In addition, these proteins have been identified to have diverse functions such as cross-talking with different kinases and playing a role in cellular signaling. However, only a few Rab proteins have been found to have a role in male germ cell development. The most notable functions of this process are performed by numerous testis-specific and/or germ cell-specific genes. Here, we describe a new Rab protein that is specifically expressed in male germ cells, having GTPase activity.

**Results:**

Testis-specific GTPase (TSG) is a male-specific protein that is highly expressed in the testis. It has an ORF of 1593 base pairs encoding a protein of 530 amino acids. This protein appears in testicular cells approximately 24 days postpartum and is maintained thereafter. Immunohistochemistry of testicular sections indicates localized expression in germ cells, particularly elongating spermatids. TSG has a bipartite nuclear localization signal that targets the protein to the nucleus. The C-terminal region of TSG contains the characteristic domain of small Rab GTPases, which imparts GTPase activity. At the N-terminal region, it has a coiled-coil motif that confers self-interaction properties to the protein and allows it to appear as an oligomer in the testis.

**Conclusion:**

TSG, being expressed in the male gonad in a developmental stage-specific manner, may have a role in male germ cell development. Further investigation of TSG function *in vivo* may provide new clues for uncovering the secrets of spermatogenesis.

**Electronic supplementary material:**

The online version of this article (doi:10.1186/s12864-016-3145-9) contains supplementary material, which is available to authorized users.

## Background

Testicular development is marked by the development and differentiation of Sertoli cells, Leydig cells and germ cells. These developmental processes are strictly regulated by a well-coordinated program of gene expression [1–3]. During Spermatogenesis, the genetic information from male germ stem cells undergoes editing and reorganization and is finally distributed into spermatozoa. The most notable functions of this process are performed by numerous testis-specific and/or germ cell-specific genes [4, 5]. Thus, identification and further characterization of testis- and germ cell-specific genes contribute greatly to our knowledge of spermatogenesis.

Rab GTPases, the largest family of small GTPases, are known as master regulators of intracellular membrane traffic [6, 7]. In humans, this family is made up of approximately 70 members, subdivided into 44 subfamilies [8, 9]. Rab proteins are present in all compartments of endomembrane system such as Golgi and endoplasmic reticulum, the nucleus, the plasma membrane, mitochondria and centrioles. In addition to their role in the extraordinary complex membrane trafficking circuit, the characterization of the Rab GTPases has revealed their diverse functions [10–12]. For example, Rab proteins have been identified to cross talk with different kinases and play a role in cellular signaling. Rab4, Rab5, Rab25 and Rab11 [13–17] have been associated with phosphoinositide kinases, Rab8 have been associated with germinal center kinases (GCKs) [18], Rab13 and Rab32 have been associated with Protein kinase A (PKA) [19, 20], and Rab2 has been associated with protein kinase C (PKC) [21]. Rab proteins have also been reported to have other functions, including nuclear signaling (Rab5, Rab8, Rab24 and others) [22, 23], regulation of mitochondrial fission (Rab32) [20], regulation of cell-matrix and cell-cell adhesion (Rab4a, Rab8b, Rab13 and Rab21) and involvement in cell growth and division or apoptosis (Rab6a, Rab11, Rab12, Rab23, Rab25, Rab35, Ran and others) [10, 24–29].

Among the Rab family of proteins, only a few are reported to be expressed in the testis. Small GTPase Rab12 is highly expressed in rat Sertoli cells and aids in the transport of vesicles from cellular fringe to perinuclear centrosome region [26]. Rab proteins have also been identified to have an important role in sperm development. Rab3A, a monomeric GTP binding protein, is expressed in the acrosomal membrane of mouse sperm and regulates zona pellucida-induced acrosomal exocytosis [30]. TBC1D9, a Rab GTPase accelerating protein (GAP), is abundantly expressed in spermatocytes and is reported to interact with Rab5, Rab7 and Rab9 [31]. Recently RabL2 has been shown to be essential for sperm tail function and male fertility [32]. In addition, Rab8B is known to assist in junction dynamics of the testis [33].

In the course of cloning androgen-induced genes from murine testis, we identified a gene encoding a protein with GTPase activity, which is likely a variant of the mouse homolog of human RASEF [34]. The expression of this gene was testis-specific; thus, it was named *Tsg* (testis-specific GTPase). TSG has a well conserved Rab domain which provides GTPase activity and classifies it among the members of the Rab/Ras family. Very limited numbers of Rab proteins have been associated with the testis. Thus, finding and characterization of a testis-specific Rab can aid our understanding of testicular development and spermatogenesis.

## Methods

### Plasmids

The mammalian expression vectors for full-length TSG, pCDNA3-HA-TSG and pCDNA3-FLAG-TSG were cloned by PCR amplification. For pCDNA-FLAG-coiled-coil, an internal XbaI site was used and the N-terminal 176 amino acids were cloned, which included the coiled-coil region from amino acid position 5 to 154. For the coiled-coil deletion clone, the N-terminal 176 amino acids were deleted, and the remaining residues were cloned into pCDNA-FLAG vector. For antibody production, the PstI-digested fragment from residue 213–523 was cloned into the pRSET-C at the PstI site. For immunocytochemistry and immunoprecipitation, either full-length or ΔN-terminal (Δ1-212) TSG was cloned into EGFP-C1 (Clontech).

### Screening of λ-testis cDNA library

A mouse testis cDNA library in Lambda ZAP was purchased from Stratagene Inc. (La Jolla, CA, USA). Screening of 2.5 × 10^5^ clones was done according to the manual. Briefly, the phage particles were immobilized on nylon membranes and then denatured in 0.5 M NaOH and 1.5 M NaCl. They were then neutralized in 0.5 M Tris (pH 8.0) and 1.5 M NaCl. Following UV cross-linking, the filters were prehybridized and then hybridized at 42 **°**C in the presence of 5X standard saline citrate (SSC), 10 % dextran sulfate, 1 mM EDTA, 50 % formamide, 10 mg/ml denatured salmon sperm DNA along with a random-primed [^32^P]-dCTP-labeled mouse TSG cDNA probe. Filters were then washed and exposed for 36 h on Kodak X-ray film at −70 **°**C. Secondary screening was performed to isolate single, pure phage plaques. Phase particles were used to recover the cDNA inserts as plasmids in the pBSαSK vector by *in vivo* excision [35].

### Cell culture

Cos-7 and 293T cells were maintained in Dulbecco’s minimum essential medium supplemented with 10 % FBS and antibiotics. All cells were cultured at 37 °C under an atmosphere of 5 % CO_2_.

### Antibody production and purification

pRSET-C TSG was transformed into BL-21 competent cells. Protein expression was induced using IPTG (Isopropyl β-D-1-thiogalactopyranoside) and then purified using Ni-affinity chromatography. Eluted protein was submitted to Abfrontier (Gwangju, Republic of Korea) for antibody production in rabbits. Serum received from the company was affinity-purified using antigen immobilized on nitrocellulose membranes as previously described [36].

### *In vitro* translation

TNT-coupled transcription-translation system (Promega) was used to perform *in vitro* translation reactions using [^35^S]-methionine according to the manufacturer’s instructions. Samples were analyzed by SDS PAGE and developed by autoradiography [37].

### Northern blot analysis

Total RNA was prepared from dissected tissues of C57BL/6 mice using Tri reagent (Molecular Research Center, Inc.). Total RNA (20 μg) run in a 1.2 % denaturing agarose gel was transferred to Zeta probe nylon membrane (Bio-Rad) and immobilized by UV cross-linking. Hybridization of membrane was done with random-primed α-[^32^P]-labeled TSG cDNA probes as described previously [38].

### Semi-quantitative reverse transcription polymerase chain reaction

For RT-PCR, 2 μg of total RNA was reverse transcribed and PCR amplified using *Tsg* specific primers. As internal control, β-actin-specific primers were used. The oligonucleotide sequences were as follows: forward 5’-AGGGGCTTTTCCTGACAACT-3’ and reverse 5’-ATTTCCAGATCCCGTTCAGA-3’ for *Tsg*; forward 5’-GAGACCTTCAACACCCCAGCC-3’ and reverse 5’-CCGTCAGGCAGCTCATAGCTC-3’ for β-actin.

### Western blot analysis

TSG-transfected 293T cells were harvested using RIPA cell lysis buffer containing protease inhibitors. Total protein was separated by SDS-PAGE and transferred to nitrocellulose membrane (Whatman Protran) as described previously [39]. Western blot analyses were performed with purified anti-TSG antibody. The signals detection was done with an ECL kit (Amersham Pharmacia). For mouse tissue samples, total lysates from dissected tissues were prepared in RIPA cell lysis buffer as indicated.

### Immunohistochemistry

Adult mouse testis was fixed in Bouin’s solution and embedded in paraffin. For immunohistochemistry, 5 μm sections were processed using the Histostain-Plus kit (Zymed Laboratories Inc.), according to the manufacturer’s instructions. Briefly, deparaffinized and rehydrated slides from mouse testes sections were heated to boiling temperature in citrate buffer at pH 6.0 and then maintained at sub-boiling temperature for 10 mins for antigen retrieval. These slides were processed with anti-TSG antibody, followed by the secondary antibody conjugated with Alexa 488 (Molecular Probes, Eugene, Oregon) for immuno-fluorescence as described [40]. TO-PRO-3 (Molecular Probes) was used to stain the nuclear DNA. Slides were examined under a laser scanning fluorescent microscope (Leica TCS SPE, Heerbrugg, Switzerland). For nuclear localization, GFP-tagged full-length TSG, ΔN TSG and GFP were transfected into Cos-7 cells cultured on gelatin coated coverslips, which were then fixed with 4 % paraformaldehyde and washed twice using PBS. The fixed cells were treated with 1 % SDS in PBS (Phosphate buffer saline) for 5 mins and then washed three times with PBS. Washed cells were incubated for 30 min with TO-PRO-3 at a 1:500 dilution in PBS and then washed twice with PBS. Coverslips were mounted on slides and analyzed with a laser scanning fluorescent microscope.

### Co-immunoprecipitation

To investigate the interaction between coiled coil motifs, 293T cells were transfected with 2 μg of each plasmid, pCDNA control, pCDNA FLAG-TSG, pCDNA HA-TSG, pCDNA-FLAG coiled-coil and pCDNA-FLAG deleted coil for 48 h. Cells were lysed with RIPA buffer (50 mM TRIS pH 8, 150 mM NaCl, and 1 % NP-40) containing protease inhibitors. Whole-cell lysate (500 μg) was incubated with 2 μg of anti-HA (sc-7392X, Lot # K0912) or anti-FLAG antibody (F7425, Sigma) overnight at 4 °C and incubated for an additional 2 h after adding 20 μl of Protein A/G PLUS-agarose beads (Santa Cruz Biotechnology). Agarose beads were washed with IP wash buffer (40 mM HEPES (pH 7.7), 0.01 % NP-40, 0.2 mM EDTA, 5 mM MgCl_2_, 150 mM NaCl and 20 % glycerol) containing protease inhibitors at 4 °C as previously described [41]. Bound proteins were analyzed using 10 % SDS-PAGE and subsequent immunoblotting using anti-HA or anti-FLAG antibodies. For immunoprecipitation of actin-bound TSG, GFP or GFP-TSG, transfected Cos-7 cells were lysed and immunoprecipitated using anti-GFP antibody and further immunoblotted using anti-actin (sc-1616) or anti-GFP antibody (sc-8334).

### GTPase assay

Cos-7 cells transfected with pEGFP or pEGFP-TSG were lysed and immunoprecipitated using anti-GFP antibody. Beads were washed with IP lysis buffer and then with TMD buffer (50 mM Tris, pH 7.4, 10 mM Mgcl2, 1 mM DTT). Collected beads were suspended in 100 mM glycine and mixed. Supernatant was collected and neutralized with 0.1 volume of Tris base and mixed with 0.1 volume of 10X PBS. The eluted protein was incubated with 4 μCi γ-P^32^ in an equal volume of TMD buffer. After 24 h, the reaction was stopped by adding 2 μl of 4 % SDS. Samples were purified by phenol:chloroform extraction and eluted in a volume of 40 μl. The samples were subsequently mixed with an equal volume of water-saturated ether and allowed to stand for 5 min, followed by collection of the lower phase and lyophilization by speed vac. The dried sample was dissolved in 2 μl of sterile distilled water and then spotted on TLC plates. Signals were detected by autoradiography.

## Results

### Chromosomal organization of TSG

**Fig. 1:**
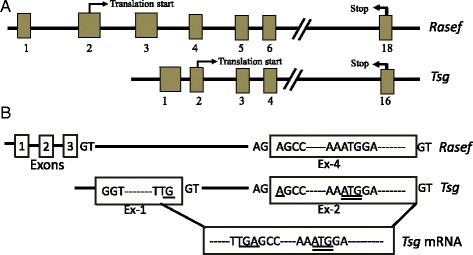
Exonic organization of *Rasef* and *Tsg.* The size of boxes corresponds to the size of exons, while the space between the boxes corresponds the introns for the genomic organization of predicted mouse *Rasef* (top) and *Tsg* (bottom) (**a**) Representation of the first two exons of *Tsg* in comparison with the first four exons of *Rasef*. The in-frame upstream stop codon and the start codon for *Tsg* are underlined with single and double lines, respectively (**b**)

*Tsg* is present on chromosome 4 in the mouse genome. It consists of 16 exons, and the translation start site lies in the 2^nd^ exon, while the stop codon is found in the 16^th^ exon (Fig. 1a). The mRNA has an open reading frame of 1593 base pairs, encoding a protein of 530 amino acids. The TSG protein shares 100 % identity with a part of RASEF in mouse (NP_001017427.1), the predicted homolog of human RASEF. RASEF is also known as Rab45, and belongs to the Rab family of GTPases. The main difference between the predicted mouse RASEF and TSG lies in the size of mRNA. Mouse *Rasef* is predicted to generate an mRNA of 5183 base pairs, which encodes a protein of 627 amino acids, while the *Tsg* has an open reading frame (ORF) of 2159 bp spanning from nucleotides 257 to 1849, which encodes a protein of 530 amino acids (Additional file 1). The marked difference lies within the N-terminal 97 amino acids of the predicted RASEF, which is missing in TSG. This difference is due to the use of an alternative transcription initiation site and the alternative exon 1 of *Tsg* mRNA, which results in translation initiation at an internal Met codon of RASEF, thereby restricting the size of protein (Fig. 1b). TSG shares more than 80 % identity with the human RASEF, while the expression of RASEF has not yet been confirmed in mouse.

### Expressional analysis of TSG

**Fig. 2:**
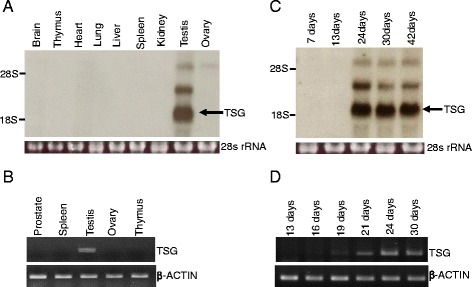
Expression analysis of TSG in mouse tissues and developing testis. Total RNAs used in northern blot analysis were isolated from various tissues of adult mice and 28S rRNA was used as a loading control (**a**) Expression of *Tsg* is specific to the testis as analyzed in different tissues of 6-week-old mice by RT-PCR. β-actin was used as an endogenous control (**b**) Total RNAs from the indicated age of developing testis were used for the analysis (**c**) Expressional analysis of *Tsg* in the indicated aged testis by RT-PCR (**d**)

The expression of *Tsg* was examined in different tissues by northern blot analysis (Fig. 2a). Expression was confined to the testis with no expression in somatic tissues or ovary. Tissue level RT-PCR analysis also confirmed the male specificity with expression confined to the testis (Fig. 2b). Northern blotting revealed two larger transcripts that hybridized with the cDNA probe of *Tsg*, which could be either *Tsg* mRNA precursors, variants of *Tsg* mRNA or homologous transcripts including mouse *Rasef*. The expression of TSG in the testis is developmentally regulated, appearing approximately 24 dpp (Fig. 2c). The major change during this period in testicular development is the appearance of haploid round spermatids which appears approximately 20 dpp [42]. RT-PCR analysis confirms the appearance of the *Tsg* transcript at 19 ~ 21 dpp, which continues to increase with development (Fig. 2d).

**Fig. 3:**
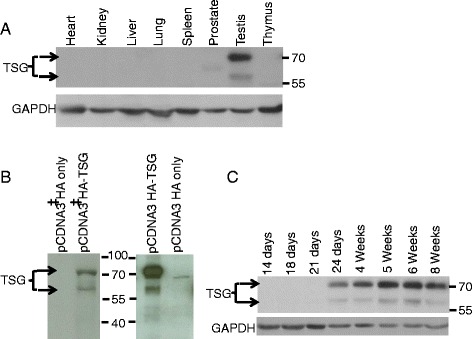
Expression of TSG protein in mouse tissues and developing testis. TSG exists as a higher molecular weight form in the testis (**a**) *In vitro* translation using radioactively labelled methionine clearly shows two forms of TSG produced from the cDNA clone after autoradiography. Similar forms of TSG were detected using anti-TSG antibody in immunoblot analyses using the lysates of 293T cells transfected with HA-tagged TSG. (‡) stands for samples of *in vitro* translation using radioactive methionine. Empty pCDNA HA vector was used as a negative control (**b**) TSG expression during testis development (**c**)

**Fig. 4:**
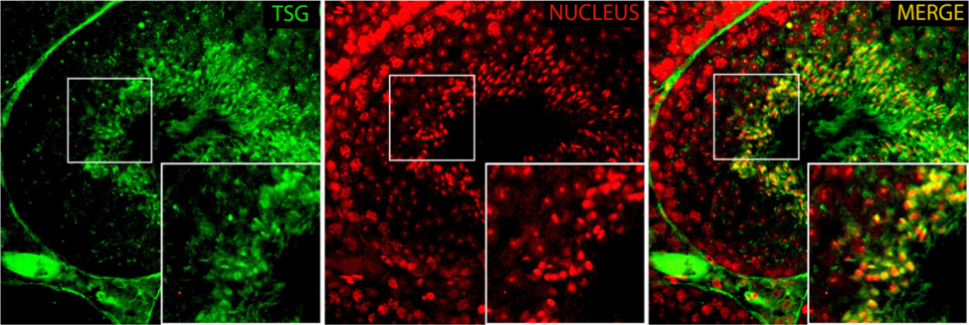
TSG is highly expressed in elongating spermatids. Immunofluorescence of the adult testis sections of mice showing stage VII tubule with Alexa 488 anti-TSG antibody (GREEN; left), TO-PRO-3 (RED, middle) and merge (right). Inset represents a higher magnification

At the protein level, TSG shows a similar expression pattern as the RNA. The expression is specific to the testis, but with a higher molecular weight than expected (Fig. 3a). To verify the size of TSG, we performed *in vitro* translation using a cDNA clone. The result confirmed two different sizes of the protein: the expected size of approximately 58 kDa and a second of approximately 72 kDa (Fig. 3b, left panel). Similar sized proteins were visualized in the lysate of TSG-overexpressing 293 T cells, which was immunoblotted using purified TSG antibody (Fig. 3b, right panel). The expression of TSG in the testis was detected at 24 days (Fig. 3c). Furthermore, immunohistochemistry was performed to analyze the expression of TSG in the testis. The immunofluorescent localization confirmed expression, mainly confined to germ cells, particularly in elongating spermatids (Fig. 4). TSG transcripts seem to be under translational regulation because the messages are expressed earlier than protein detection, at 19 ~ 21 dpp when round spermatids are developing. Due to the limited transcriptional activity in elongating spermatids, messenger RNAs for many proteins expressed in elongating spermatids are actually produced in round spermatids and undergo translational repression until development has reached the elongating spermatid stage [43].

### Domain analysis

**Fig. 5:**
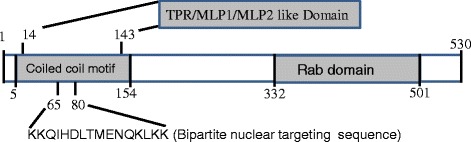
Structural organization of TSG protein. The N-terminal region harbors the coiled-coil motif and the C-terminus contains the Rab domain. Bipartite nuclear targeting sequence is located in the middle of coiled-coil motif. The coiled-coil region also harbors the TPR/MLP1/MLP2-like domain

Architecture domain analysis of TSG using the SMART database (http://smart.embl-heidelberg.de/) revealed a coiled-coil motif present in the N-terminus of TSG (Fig. 5). Further motif scan analysis (http://myhits.isb-sib.ch/cgi-bin/motif_scan) ascertained the presence of a bipartite nuclear targeting sequence, suggesting that TSG is a nuclear-targeted protein. The coiled-coil motif extends from amino acid residues 5 to 154, with the nuclear targeting sequence embedded extending from residues 65 to 80. The coiled-coil motif region also harbors a TPR/MLP1/MLP2-like domain, which is a feature of myosin-like proteins. The C-terminus of TSG contains the Rab/Ras GTPase domain, which is evolutionarily conserved.

### Self-interaction of TSG through the coiled-coil motif

**Fig. 6:**
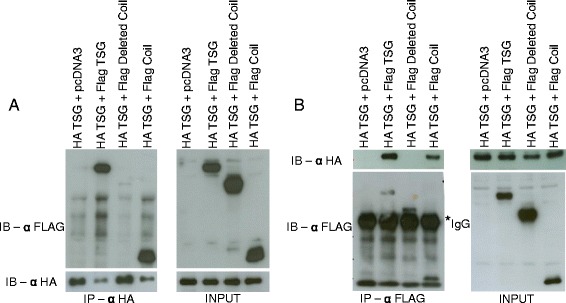
TSG is able to self-interact through the coiled-coil motif. Immunoprecipitation using anti-HA antibody (**a**) or anti-FLAG antibody (**b**) The immunoprecipitates were analyzed by western blotting using anti-FLAG and anti-HA antibodies. All HA- and FLAG-tagged constructs were cloned into the pCDNA3 vector, and 5 % of the total protein was taken as input. The asterisk (*) represents the non-specific IgG

TSG is detected at a higher molecular weight than expected in the testis (Fig. 3b). Therefore, we sought to determine whether TSG is capable of undergoing self-interaction and, if so, whether the coiled-coil motif of TSG is involved in that interaction. To investigate this, we prepared full-length TSG constructs tagged with FLAG or HA and TSG deletion mutants lacking the coiled-coil motif or containing only the coiled-coil motif. HA-tagged full-length TSG was expressed with FLAG-tagged full-length or deletion mutants of TSG in 293T cells, and the cell lysates were subjected to immunoprecipitation using anti-HA (Fig. 6a) or anti-FLAG antibody (Fig. 6b). FLAG-tagged full-length TSG as well as the isolated coiled-coil domain interacts with HA-tagged full-length TSG, while the TSG deletion mutant lacking the coiled-coil motif loses the ability to interact with full-length TSG. These results show that TSG is able to form multimers through the coiled-coil motif and suggest that this property may be the reason for the higher than predicted molecular weight form of TSG.

### TSG is a nuclear protein

**Fig. 7:**
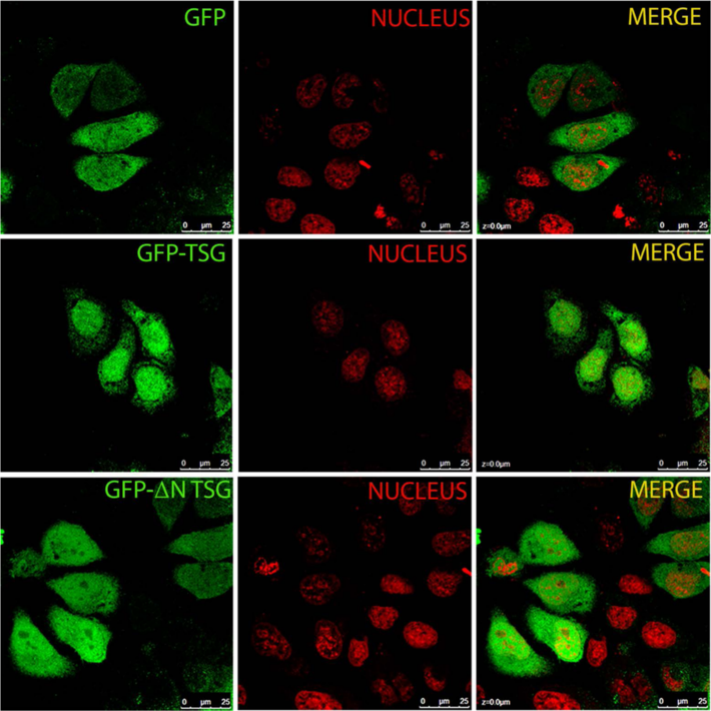
TSG is likely a nuclear protein. GFP-tagged TSG expressed exogenously in HeLa cells was concentrated predominantly in the nucleus (Green) while N-terminus-deleted TSG was localized to the cytoplasm similar to GFP (Green) only. Nuclear DNA was stained using TO-PRO-3 (Red)

TSG contains a nuclear localization signal (NLS). In addition, immunohistochemical analysis of TSG expression in the testis showed that TSG is localized to the elongating spermatids (Fig. 4). Therefore, we validated the functionality of the NLS using a TSG deletion construct, lacking the most N-terminal region containing the NLS. The N-terminus-deleted GFP-tagged TSG clone was restricted from the nucleus and remained distributed in the cytoplasm, while the full-length GFP-tagged protein was localized to the nucleus (Fig. 7).

### TSG has GTPase activity

**Fig. 8:**
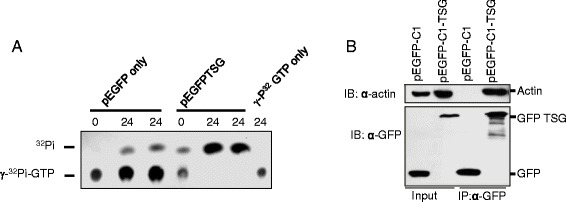
Functional characteristics of TSG. TSG has GTPase activity. The extract from Cos-7 cells transfected with pEGFP or pEGFP-TSG was incubated with γ- [^32^P]-GTP for 24 h and then subjected to TLC (**a**) TSG binds to actin inside the cell. Cos-7 cells were transfected with GFP only or with GFP-TSG, and lysates were immunoprecipitated with anti-GFP antibody. A total of 10 % of the sample was loaded as input (**b**)

Proteins containing the Rab domain have been shown to have GTPase activity. TSG contains a Rab domain at the C-terminus; thus, we expected it to display GTPase activity. To verify the GTPase activity, pEGFP only or pEGFP-TSG fusion protein constructs were expressed in Cos-7 cells, and the cell lysates were immunoprecipitated using anti-GFP antibody. The eluted protein was incubated with γ-[P^32^]-labeled GTP for 24 h. The GFP-tagged TSG released the γ-[P^32^] from GTP, which was detected by thin-layer chromatography (Fig. 8a).

### Myosin-like property

Myosin is a motor protein that utilizes the energy from ATP hydrolysis to move along the actin filament. TSG has a TPR/MLP1/MLP2-like domain, which is a feature of myosin-like proteins. To confirm the myosin-like property, we performed a co-immunoprecipitation experiment using Cos-7 cells transfected with GFP-tagged TSG or GFP vector only. The cell lysate with GFP-TSG expression was immunoprecipitated with anti-GFP antibody, and GFP-TSG was co-immunoprecipitated with actin protein, while no such interaction was seen in the lysate expressing only GFP (Fig. 8b). These results indicate the possibility for TSG to behave like myosin protein.

## Discussion

Several Rab proteins and Rab effectors have been reported to form oligomers and the self-interacting property of these proteins are important for their function. For example, RILP forms a homodimer to interact with Rab7, and disruption of dimerization abolishes its interaction with Rab7 [44]. A family of Rab11-interacting proteins (FIPs) has also been reported to have the ability to self-interact [45–47]. Human RASEF has been characterized as a self-associating GTPase localized in perinuclear area of cells [34]. The mouse homologue of RASEF has not yet been verified, but the predicted protein reported in the NCBI database is completely identical to TSG except for an additional 90 amino acids located at its N-terminus and thus is predicted to self-interact as shown for TSG. As per our analysis, TSG is highly expressed in the testis and is a nuclear localizing GTPase because deletion of the bipartite nuclear localization signal restricts its localization to the cytoplasm. Conversely the human RASEF is ubiquitously expressed and localized to the membrane. Both of these proteins share a Rab domain at the C-terminus and thus have GTPase activity.

Proteins with coiled-coil motifs typically have a role in membrane traffic and have functionally distinct domains at their N- or C-terminus [48]. Rabaptin5, a large coiled-coil protein, has a distinct N and C terminal domains that binds to Rab4 and Rab5 respectively [49]. Rab45, also called human RASEF, has an EF-hand domain at the N-terminus, and a Rab homology domain in the C-terminus [34]. In the same manner, the predicted mouse RASEF has an EF-hand domain as well as a Rab domain, but TSG lacks the EF-hand domain, which makes it different from RASEF. Mouse RASEF, if verified, may show membrane localization similar to the human RASEF. However, we cannot rule out the possibility that mouse RASEF is a nuclear-targeted protein because the bipartite nuclear localization sequence found in TSG is also present in the predicted RASEF protein, while human RASEF completely lacks this sequence.

Human RASEF is a 740 amino acid protein with an expected molecular weight of approximately 81 kDa, but the wild type clone expressed in HeLa cells was actually observed to be approximately 100 kDa [34]. Similarly, TSG is a 530 amino acid protein and is expected to have a size of 58 kDa, but it appears as a mixture of both 72 kDa and 58 kDa; the reason might be its self-interacting property. When we checked the protein expression level in mouse testis, the majority of the protein appears as the high molecular weight form, while a very small, almost undetectable, portion of the expected size is observed. This suggests true functionality of this protein in the testis because most coiled-coil proteins need to self-interact and form oligomers to be functional. Such self-interacting proteins lose their function when the interaction is abolished [44]. The appearance of this protein in the elongating spermatids after three weeks of murine testis development indicates its importance in later stages of germ cell development. In the course of germ cell development in the testis, round spermatids appear around 20 dpp and the condensing/elongated spermatids start to appear around 24 dpp. The final spermatozoa are released at 35 dpp [42].

Myosins consist of ATP-dependent proteins that are involved in a wide range of cell motility processes as well as muscle contraction. Heavy chains of Myosin have three chiseled regions: the motor, neck, and tail domains [50]. The motor domain binds to actin in an ATP-dependent manner [51, 52]. The neck domain accords the binding of light chains and assists as a lever arm to permit movement of motor domain [51]. The tail domain is the most variable region of myosins with various lengths and functions depending on the motifs present in its sequence, such as coiled-coil dimerization regions, MyTH4, FERM, or SH3 domains for protein–protein interactions [51]. TSG does not have the head region of myosin but it still interacts with actin, which suggests TSG may be part of complex which functions as a GTPase protein. GTPases are known to complex with myosin [53, 54]. The rat myosin protein myr5 has been described as a GTPase activation protein *in vivo* [55]. TbRab23 is a nuclear-associated Rab protein that renders stability to nuclear structure [56]. There are recent reports identifying multiple Rab proteins that interact with myosin Va. Lindsay et al. screened all human Rab proteins for the ability to bind myosin Va and revealed an interaction with Rab3B, 3C, 6A, 6A´, 6B, 11B, 14, 25 and 39B [57]. This report adds to the previously reported interactions of Rab3A, 8A, 10, 11A and 27A with myosin Va [58–61].

## Conclusion

In the present study, we have elaborated the expression and localization of TSG, a protein with GTPase activity and self-interacting properties capable of forming oligomers. The germ cell-specific expression of TSG makes it an attractive candidate to address its biological role during testicular germ cell development.

## Additional file

Additional file 1: Nucleotide and amino acid sequences of TSG. Complete mRNA sequence and the deduced amino acid sequence identified for TSG having the start and the stop codon underlined and bolded. (DOCX 13 kb)

## Abbreviations

Dpp: Days postpartum
GAP: GTPase accelerating protein
GCKs: Germinal center kinases
IPTG: Isopropyl β-D-1-thiogalactopyranoside
NLS: Nuclear localization signal
ORF: Open reading frame
PBS: Phosphate buffer saline
PKA: Protein Kinase A
PKC: Protein kinase C
Rab: Ras-related proteins in brain
RASEF: RAS and EF hand domain containing
SSC: Standard saline citrate
TSG: Testis-specific GTPase
