# Supplementary material for: Testis-Specific GTPase (TSG): An oligomeric protein
Source: BMC Genomics. 2016 Oct 10;17:792. doi: 10.1186/s12864-016-3145-9 (PMC5057473; doi:10.1186/s12864-016-3145-9)
Supplement: Additional file 1: — Nucleotide and amino acid sequences of TSG. Complete mRNA sequence and the deduced amino acid sequence identified for TSG having the start and the stop codon underlined and bolded. (DOCX 13 kb) [file 12864_2016_3145_MOESM1_ESM.docx]

>Complete mRNA (TSG)

GGCACGAGGTAGATGGTTTCCATCCTATGAGGTCAACTCCTCAGAACTGCTGAGTCCATCCTGAAAAATTCAGAATGTTTTTTTTCACCAGCTACTTGACAAAAACAGATGCTAGGGGCTTTTCCTGACAACTAGTTCACTGAGGAAGGCAGTGAAGTGGGGATATGGAGTTGGTGATCAGGGCCAATGCTGTTTTACCCTTGGCAATTCTTCTGTTGAGCCCAGGACAAGGCGGCCATTCAGCTAAGTGAGCTGGAAGAGGAA**ATG**GATCAGAGGATTCAAGCTGTGGAAAATGAGTCCCGGAAAGATGAAAAGCGCAAAGCTGAGGAGGCTCTCACTGACCTCAGGCGTCAGTATGAAACAGAAGTAGGAGACTTACAAGTGACCATTAAAAGGTTAAAAAAGCTTGAAGAGCAATCAAGACAAATAAGTCAAAAGCAAGATGTGACGGCATTGAAGAAGCAAATCCATGATTTAACAATGGAAAATCAAAAACTCAAGAAAGAACTTTTGGAAGCACAGACAAACGTAGCCTTCCTTCAGAGCGAACTAGATGCTTTGAAAAGTGATTATGCTGACCAGAGTCTGAATTCTGAACGGGATCTGGAAATAATCCGAGAATACACAGAAGATCGAAGCAGTCTTGAGAGGCAAATTGAAATCCTCCAAACAGCTAACCGGAAGCTGCATGACAGTAATGATGGCCTCAGGAGTGCACTGGAAAACACTTACAGCAAGCTCAACAGATCCTTGCGTATAAATAACATATCTCCAGGGAATACAATTTCTAGAAGCAGTCCCAAATTTAATCATCATTCCTCTCAGCCACTGGCCTATGACAGGTCATTCCATTCTTCATATGCAGATGAGGATTGTGACTCTTTGGCTCTCTGTGACCCTCTGCAGAAGATGAATTATGAAGTTGACAGCCTGCCTGAAAGCTGTTTTGACAGCGGTCTGTCTACTCTGAGAGACAATGAGTGTGACTCCGAGGTGGACTACAAGCATCAGGGAGAATTTCAGACTTTGCACAGAACAGAGGAGAGCCTTGGGGGCGATGCATCAGACACAGATGTTCCAGATATAAGGGATGAAGAAGCGTTTGATTCTGAAAGTGTGGCCTCTGTCTTACACTGGCAGCCCCAGGGCTCTGCTGGTGAGGGCAGCACTCTTAGTTCCTCCAGAAAGCCCATCTCAGCTCTTTCACTTCAGACAGACATGGTGGATAACACCTCCAAGGTCACATCTCAGAAGGCTTACAAGATTGTGCTGGCTGGCGACGCCGCAGTGGGAAAGTCCAGCTTCCTCATGCGTCTCTGCAAGAATGAGTTTCAGGGGAACACAAGCGCAACTCTAGGAGTTGATTTCCAAATGAAAACTCTGATTGTGGATGGTGAACAAACAGTTCTTCAGCTCTGGGATACAGCTGGGCAAGAGAGATTTAGGAGTATCGCCAAGTCTTACTTCCGAAAAGCAGATGGTGTCTTACTGCTATACGATGTTACTTGTGAGAAAAGCTTTCTTAACGTCCGAGAATGGGTGGATATGGTTGAGGATGGAACCCACAGAACTATTCCTATCATGTTAGTAGGAAACAAGGCTGATCTTCGTGATGTTGATAATGCAGAGAATCAAAAGTGTATATCAGCATATCTTGGAGAAAAACTGGCCATGACCTATGGGGCATTATTCTGTGAAACAAGTGCCAAAGATGGCTCCAACGTGGTGGAAGCTGTTCTCCATCTTGCACGGGAAGTGAAGAAAAGGACAGAGGATGACGACAGCAGATCTATCACCAGTCTGGCTGGGTCTACTTCTAAAAAGTCACTGCAGATGAAAAACTGTTGCAATGGC**TAA**AGCCCAGTTGTTTTGTGTTCACAAAGTCCTTGCTTCCAGATTACTGACTGTGGGACCCTGGCTCTCCATGAAGCAGTGCATAATATGACACTGGGATTTGAGGAATCACAGCCTGGCCAATTGCACTGTTTTTCCCCGCTCTCAGCAACCTGAAGCTTGCTTTGTTCTATCTCAGTGAGCTATTTGGGATGGCTGGCCCTATAGATCTAAGAGAAAATGTGTTAATGTTTTTAATATGATATGGTAAAATAAAATAACTTTTTCAGAAAAAAAAAAAAAAAAAAAAAAAAAAAAAAAAAAAAAAAAAAAA

>TSG protein

MDQRIQAVENESRKDEKRKAEEALTDLRRQYETEVGDLQVTIKRLKKLEEQSRQISQKQDVTALKKQIHDLTMENQKLKKELLEAQTNVAFLQSELDALKSDYADQSLNSERDLEIIREYTEDRSSLERQIEILQTANRKLHDSNDGLRSALENTYSKLNRSLRINNISPGNTISRSSPKFNHHSSQPLAYDRSFHSSYADEDCDSLALCDPLQKMNYEVDSLPESCFDSGLSTLRDNECDSEVDYKHQGEFQTLHRTEESLGGDASDTDVPDIRDEEAFDSESVASVLHWQPQGSAGEGSTLSSSRKPISALSLQTDMVDNTSKVTSQKAYKIVLAGDAAVGKSSFLMRLCKNEFQGNTSATLGVDFQMKTLIVDGEQTVLQLWDTAGQERFRSIAKSYFRKADGVLLLYDVTCEKSFLNVREWVDMVEDGTHRTIPIMLVGNKADLRDVDNAENQKCISAYLGEKLAMTYGALFCETSAKDGSNVVEAVLHLAREVKKRTEDDDSRSITSLAGSTSKKSLQMKNCCNG
